# Incontinentia pigmenti in a child with suspected retinoblastoma

**DOI:** 10.1186/s40942-017-0088-5

**Published:** 2017-09-18

**Authors:** Stephanie J. Weiss, Archana Srinivasan, Michael A. Klufas, Carol L. Shields

**Affiliations:** 1 Ocular Oncology Service, Suite 1440, Wills Eye Hospital, 840 Walnut Street, Philadelphia, PA 19107 USA; 2 Ophthalmology Department, Drexel University College of Medicine, Philadelphia, PA USA; 3 Mid Atlantic Retina, The Retina Service of Wills Eye Hospital, Thomas Jefferson University, Philadelphia, PA USA

**Keywords:** Eye, Incontinentia pigmenti, Bloch–Sulzberger syndrome, Retinoblastoma, Pseudoretinoblastoma, Retinal detachment

## Abstract

**Background:**

Incontinentia pigmenti is a rare X-linked dominant syndrome caused by mutation in the NEMO/IKKgamma gene, and characterized by a spectrum of cutaneous, ocular, neurologic and dental abnormalities. In the eye, findings include retinal vascular non-perfusion, occasionally with traction retinal detachment, retinal fibrosis, and retinal pigment epithelium defects. These findings can resemble retinoblastoma, especially when vitreoretinal fibrosis produces leukocoria.

**Case report:**

A 2-month-old girl born full-term presented with leukocoria, suspicious for retinoblastoma. She was found to have an ischemic retrolental fibrovascular retinal detachment. In addition, there was linear cutaneous hyperpigmentation, diagnostic of incontinentia pigmenti.

**Conclusions:**

Retinoblastoma can be a challenge to diagnose. There are numerous simulating lesions that can present with leukocoria and retinal detachment, including incontinentia pigmenti. Recognition of the cutaneous features of incontinentia pigmenti contributes to early detection of related ophthalmologic, neurologic and dental abnormalities.

## Background

Incontinentia pigmenti (IP), also known as Bloch–Sulzberger syndrome, is a rare X-linked dominant syndrome typically lethal in males, defined by characteristic skin findings along with ocular, neurologic and dental abnormalities [1–6]. The pathogenesis of IP is attributed to a mutation in the NEMO/IKKgamma gene located at the Xq28 loci, which leads to the activation of eotaxin which stimulates accumulation of eosinophils in tissue [1–5]. In the skin, IP manifests with a staged rash consisting of erythema, vesicles and pustules at birth, followed by verrucous lesions that evolve into swirled flat hyperpigmentation along the lines of Blaschko, ultimately resulting in linear, atrophic, hypo- and hyper-pigmented streaks [2–4]. Dental abnormalities include adontia and/or pegged and widely spaced teeth [2–5]. Neurologic manifestations are varied and include seizures, mental retardation, cerebral atrophy, corpus callosum hypoplasia and hemorrhagic necrosis [1–6]. These neurologic findings are closely related to the ophthalmologic manifestations, thought to be caused by similar eosinophilic vasoocclusive phenomena [2, 4, 5].

Incontinentia pigmenti-related ocular abnormalities have been reported in 50–77% of patients, with retinal features being the most striking [1, 2, 4, 5, 7]. These findings include retinal vascular non-perfusion with pathologic neovascularization that may progress to tractional retinal detachment with retrolental fibrous tissue [1, 3–6]. Other retinal findings include foveal hypoplasia, hyperpigmentated/hypopigmentated retinal pigment epithelial (RPE) defects, and nodular RPE proliferation [1–6]. Recently, optical coherence tomography (OCT) imaging in patients with incontinentia pigmenti has shown inner and outer retinal layer thinning associated with incontinentia pigmenti [8]. Rarely, ocular abnormalities associated with IP have been reported to simulate retinoblastoma in that both can manifest leukocoria, strabismus, retinal detachment and intraocular calcification [3]. We present a 2-month-old female who presented with leukocoria, suspicious for retinoblastoma, but found to have classic cutaneous and ocular manifestations of IP.

## Case presentation

A 2-month-old Asian-Indian female born full-term at 39 weeks gestation and weighing 2.92 kg at birth was found at 6 weeks of age to have leukocoria of the right eye, suspicious for retinoblastoma. She was referred to the Ocular Oncology Service at Wills Eye Hospital for further evaluation.

**Fig. 1:**
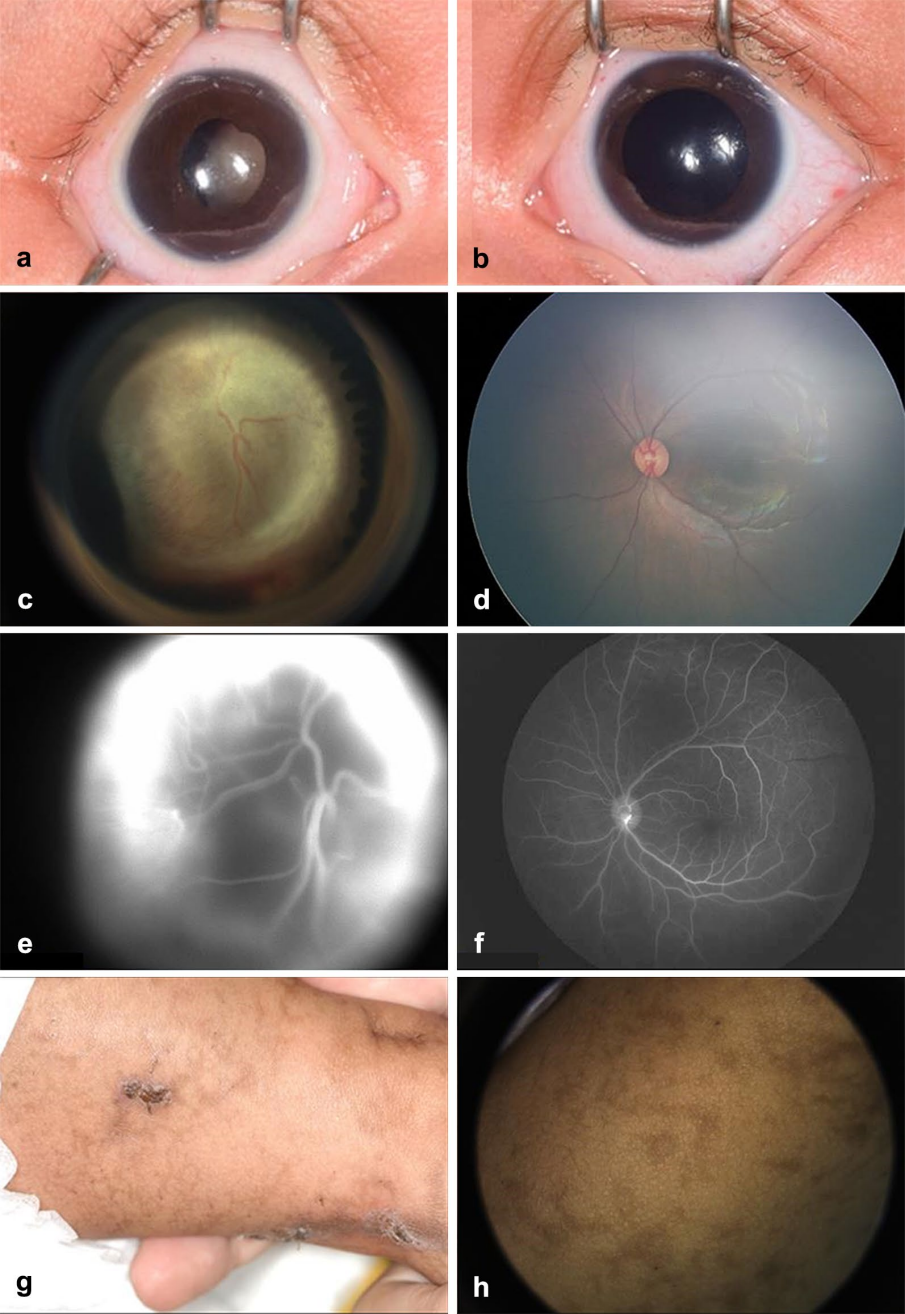
Clinical features of incontinentia pigmenti. A 2-month-old Asian Indian female was found at 6 weeks of age to have leukocoria of the right eye (**a**) with a normal appearing left eye (**b**). Fundus examination of the right eye revealed a tractional retinal detachment (**c**) behind the lens, dragging the pars plicata inward and producing vitreous hemorrhage inferiorly. The left fundus (**d**) was normal. Fluorescein angiography of the right eye (**e**) revealed marked hyperfluorescence with diffuse leakage suggestive of neovascularization and the left eye (**f**) was healthy with normal perfusion. (**g**) Involuting cutaneous vesicular lesions and (**h**) linear hyperpigmentation (lines of Blaschko) were consistent with incontinentia pigmenti

On examination, visual acuity was no fix or follow in the right eye (OD) and fix and follow in the left eye (OS). Intraocular pressures were 13 mm Hg OD and 16 mm Hg OS. The anterior segment examination revealed microcornea with horizontal diameter of 10 mm OD, scattered posterior synechiae of the iris, and dense retrolental fibrovascular membrane with anterior and centripetal displacement of the ciliary processes (Fig. 1a, b). On anterior examination, OS appeared normal with horizontal corneal diameter of 11 mm.

Fundus examination OD revealed a total closed-open funnel tractional retinal detachment with contraction posterior to the lens and anterior loop fibrovascular contraction. There was mild hemorrhage inferiorly within the vitreous cavity (Fig. 1c). Fundus examination OS was normal without evidence of peripheral traction or retinal detachment (Fig. 1d). Fluorescein angiography (FA) revealed diffuse late hyperfluorescence and leakage consistent with retinal neovascularization OD. FA OS exhibited normal arterial and venous filling with no evidence of peripheral hypofluoresence consistent with nonperfusion in late frames. There was no evidence of leakage OS (Fig. 1e, f). Magnetic resonance imaging (MRI) of the brain and orbits disclosed total retinal detachment with a decreased axial length of 17.1 mm OD compared to 18.0 mm OS. These features were suggestive of tractional retinal detachment with microphthalmia OD.

The parents denied other family members with similar findings. They indicated that the infant was found at birth to have a vesicular cutaneous rash over the entire body that spontaneously resolved. Cutaneous examination disclosed extensive linear hyperpigmented streaks along the lines of Blashko (Fig. 1g, h). Given the ocular and cutaneous features, the diagnosis of incontinentia pigmenti was suspected and confirmed on skin biopsy. The pain-free OD was managed conservatively with observation. Given the risk for future ischemia OS, close serial examination with FA was advised.

## Discussion and conclusions

Retinoblastoma typically appears as a yellow-white retinal mass, often with surrounding subretinal fluid, subretinal seeds and vitreous seeds [9]. The presenting symptoms for retinoblastoma most often include leukocoria and strabismus [9]. However, similar findings can be associated with a broad spectrum of other pediatric fundus abnormalities, leading to diagnostic uncertainty [9]. In a large study of 604 cases of pseudoretinoblastoma published by Shields et al. in 2012, the most common conditions simulating retinoblastoma and leading to incorrect diagnosis included Coats disease (40%), persistent fetal vasculature (28%), vitreous hemorrhage (5%), toxocariasis (4%), familial exudative vitreoretinopathy (3%), rhegmatogenous retinal detachment (3%), coloboma (3%), astrocytic hamartoma (2%), combined hamartoma of retina and RPE (2%), and endogenous endophthalmitis (2%) [9]. In that study, incontinentia pigmenti was reported in only 2 cases, accounting for <1% of pseudoretinoblastoma cases [9].

Due to its rarity, a high index of suspicion is often required to make an accurate diagnosis of incontinentia pigmenti. Early detection is critical to maximize visual potential and prevent retinal detachment, as well as avoid other systemic comorbidities including potentially life-threatening neurologic manifestations, such as stroke, coma, and death from brain ischemia [1–6]. Ocular abnormalities are recorded in approximately 50–77% of patients with incontinentia pigmenti, and include microphthalmia, corneal opacities, iris hypoplasia, cataract formation, optic nerve atrophy, macular hypoplasia and RPE abnormalities as well as retinal and choroidal vascular ischemia that leads to neovascularization with traction retinal detachment and vitreous hemorrhage [5, 7]. OCT in areas of non-perfusion in patients with IP has been shown to have thinning involving inner retinal layers as well as the outer plexiform layer which is present prior to laser treatment and remains stable over time indicating vascular abnormalities during the developmental process [8]. Neurologic findings are detected in approximately 30% of patients and include corpus callosum hypoplasia, seizures, mental retardation, cerebral atrophy, and hemorrhagic necrosis [4]. The ocular and neurologic findings are thought to be caused by similar vasoocclusive events, secondary to eosinophil accumulation within and around vessels [1–5]. These abnormalities are a result of a mutation in the NEMO/IKK gamma gene, found on the X chromosome, that functions as a part of the nuclear factor kappa B (NFkB) signaling pathway that controls several chemokines including eotaxin (an eosinophil chemokine) [1, 3, 5]. Mutation of this gene leads to an increase in eosinophil activation through the NFkB pathway via eotaxin as well as several cytokines including granulocyte-monocyte colony-stimulating factor and interleukin 5 [4].

In addition to the ocular and neurologic vasoocclusive phenomena, this mutation is also responsible for cutaneous and dental abnormalities associated with incontinentia pigmenti [1, 3, 5]. Reported to be found in 90–100% of patients, the staged cutaneous rash is pathognomonic for IP and progresses from a vesicular rash with pustules and erythema from birth to 2 weeks of age, followed by verrucous lesions by 2–6 weeks of age, then swirled flat hyperpigmentation along the lines of Blaschko by 12–26 weeks of age, finally leading to linear, atrophic, hypo- and hyper-pigmented streaks in adulthood [4, 9]. The rash is caused by eosinophil accumulation in intra-epidermal vesicles with significant inflammation (stage 1) which later transforms into epidermal hyperkeratosis with persistent eosinophilia (stage 2) followed by post-inflammatory melanin deposition (stage 3) and epidermal atrophy (stage 4) [4]. Despite the serious nature of the ocular and neurologic manifestations, the cutaneous findings are often first noted and strongly suggestive of the underlying diagnosis [4, 6]. Dental abnormalities, found in 80% of cases, include pegged and widely spaced teeth and adontia [4]. These typically are noted at the time of tooth eruption, and dental evaluation is recommended by age 2 years [4]. Evaluation of mother and other female siblings could reveal similar dental problems [4].

Several reports have been published describing the wide array of initial ophthalmologic findings associated with incontinentia pigmenti, but given its rarity, long term follow up of the natural progression and treatment outcomes is limited. Chen et al. published on the retinal findings in 25 patients with incontinentia pigmenti, with mean follow up of 9.3 years, and noted high risk for tractional (28%) or rhegmatogenous (16%) retinal detachment in the presence of neovascularization [1]. However, despite prophylactic laser ablation to areas of retinal vascular non-perfusion, 3 of 4 eyes went on to develop tractional retinal detachment [1]. In addition, neovascularization has been shown to remain stable or regress spontaneously in some cases, making laser photocoagulation controversial [1].

Retinoblastoma is a serious ocular malignancy in children and can be a challenge to diagnose [3, 9, 10]. There are numerous simulating lesions that can present with leukocoria and retinal detachment, particularly in a young child, including incontinentia pigmenti [3, 9, 10] The defining features of incontinentia pigmenti, the cutaneous blisters and pigmentation, contribute to early recognition and anticipation of related neurologic, dental, and ophthalmologic abnormalities.

## Abbreviations

IP: incontinentia pigmenti
RPE: retinal pigment epithelium
OCT: optical coherence tomography
OD: right eye
OS: left eye
MRI: magnetic resonance imaging
NFkB: nuclear factor kappa B
